# Updated Conversion Table for the Multiple Criteria Qualitative Value-Based Pricing Framework “MARIE”

**DOI:** 10.3390/jmahp14010012

**Published:** 2026-02-25

**Authors:** Akina Takami, Ataru Igarashi

**Affiliations:** Department of Health Policy and Public Health, Graduate School of Pharmaceutical Sciences, The University of Tokyo, 7-3-1, Hongo, Tokyo 113-0033, Japan

**Keywords:** value-based pricing, drug pricing, regulation, regulatory science, methodology, economics, pharmacoeconomics

## Abstract

**Background**: Value-based pricing has the potential to contribute to the appropriate allocation of healthcare expenditures. We developed the “MARIE”, a qualitative scheme that evaluates broad value elements without requiring a comparator to estimate new drug prices. In this study, we updated the conversion table that monetizes points calculated from value elements. **Methods**: We investigated and calculated the daily drug prices at the time of listing for drugs containing new active ingredients that were included in the National Health Insurance Drug Price List from fiscal year 2015 to 2024, summarizing the data using descriptive statistics. **Results**: New drug prices are listed annually in Japan, so we updated the conversion table using current drug prices to maintain continuity. This study also observed a trend where the median daily price tended to be higher as the maximum number of patients decreased. **Conclusions**: The MARIE method, our developed framework to qualitatively evaluate the various values of drugs, monetizing points calculated based on the elements of value via the conversion table, plays a crucial role. Updating the conversion table based on the latest data maintains continuity with the current drug pricing system and is considered to contribute to the social implementation of the MARIE method.

## 1. Introduction

### 1.1. The Medical Care System and Medical Cost in Japan

In Japan, both medical expenses and drug costs have been rising year over year due to an aging population and further advancements in sophisticated medical treatments available. National medical expenses for fiscal year (FY; April to March of the following year) 2022 reached 46.6967 trillion yen, representing a 3.7% increase when compared to 45.0359 trillion yen recorded in FY 2021 [1].

Japan operates a universal health insurance system, ensuring all citizens are enrolled in public health insurance. This allows nationwide access to receive high-quality, equitable medical care with low out-of-pocket costs [2]. Medical fees are set at pre-determined rates, and drug prices are similarly fixed as official prices. At present, approximately 20% of total medical expenses are spent on drug costs [3]. The central approach for reducing drug costs is the revision of medical fees. A scheduled revision of the price of drugs occurs every two years and plays a crucial role in controlling pharmaceutical expenditures. In addition to the biennial revision, an interim price revision was introduced in FY 2021. This has also resulted in increased savings in many drug categories.

Yet when the usage of certain greatly exceeds initial projections, their prices are significantly reduced without considering the value they provide. Such frequent and continuous price reductions also diminish the appeal of the pharmaceutical market in Japan and raise concerns about reduced motivation for pharmaceutical companies to develop new drugs. Moreover, it is considered difficult to achieve overall healthcare efficiency solely through reduction in drug cost, and an appropriate allocation of total healthcare expenditures is desired.

### 1.2. The Drug Pricing System in Japan

Drug prices in Japan are set under the Drug Price Standards, and the reimbursement price for drugs is uniform for all citizens. Under the current Japanese drug pricing system, the pricing of new drugs is determined using two methods: the cost-plus method and the similar drug comparison method [4]. With the cost-plus method, drug prices are calculated by aggregating costs such as manufacturing costs, research and development costs, operating profits, and distribution costs. Next is the similar drug comparison method, where drug prices are determined by comparing with the price of existing drugs that were calculated by the cost-plus method. Overall, whether using the cost-plus method or the similar drug comparison method, the drug pricing system can generally be regarded as one where prices are determined largely by the expense of manufacturing. Furthermore, additional premiums applied based on qualitative assessments such as usefulness, marketability, and pediatric use.

Following inclusion in the National Health Insurance Drug Price List, drug prices are revised based on market rate and market size. Furthermore, prices are reviewed through the mechanisms of re-pricing for indicational change, re-pricing for change of dosage and administration, and re-pricing for market expansion. By contrast, examples of drugs whose prices increased after launch are limited. At the time of publication, we have found six products (UFT, JARDIANCE, REPATHA, FORXIGA, CANAGLU, and PRALUENT) that have received price adjustments after confirmation of true clinical benefit and these adjustments only amount to a modest figure of increase, approximately 5% [5].

Under the current drug pricing system, two problems exist as follows: (1) when setting prices for new drugs, the price is determined based on the manufacturing and operational costs, but the resulting total does not necessarily reflect the value provided by the new drugs, creating a gap between price and value; (2) even after drugs were listed, frequent and continuous price reductions occur that do not reflect the value of the drug. While the cost-plus method and price adjustments accompanying market expansion due to healthcare budget constraints are reasonable, price adjustments that fail to reflect the value of the drug diminish pharmaceutical companies’ motivation to develop and launch new breakthrough drugs in Japan. Consequently, this risk may incentivize a strategy where companies seek indications with the fewest patients to secure higher drug prices, only later obtaining indications with larger patient populations. This could ultimately limit patient access to effective medicines.

### 1.3. Value-Based Pricing

Drug prices determined using the current drug pricing system do not necessarily reflect the various values provided by drugs. For this issue, value-based pricing (VBP) could be a potential solution. VBP has gained attention in recent years as a pricing method where pricing is based on the value a drug provides, enhancing its appeal to stakeholders. Various discussions and reports on VBP have been conducted up to now. Jommi et al. defined four operational steps for value-based pricing: identification of value domains, measurement of value, aggregation of measures, and conversion of value into prices [6].

In our previous research, we conducted a systematic review to investigate the elements of drug value and pricing methods used in VBP approaches [7]. The main selection criteria were the reporting of value elements, VBP methods, and estimated prices; eight papers met these criteria [8,9,10,11,12,13,14,15]. Of these, we identified four that had adopted the cost-effectiveness analysis (CEA) approach [8,9,10,11], while the remaining studies used different approaches [12,13,14,15]. Regarding the former approach, methods that require advanced model development (such as Markov models) are applied, which present numerous challenges in terms of the versatility and simplicity offered by these methods. Also, Lakdawalla et al. proposed 12 value elements for VBP [16].

While a price estimation method based on the broader value elements of a drug, including qualitative ones, is desirable, no specific methodology has been proposed for the value elements used in VBP and for converting the value of a drug into monetary terms. Additionally, methods based on prior comparator-based approaches suffer from issues such as being influenced by the price and characteristics of the comparator drug, so a non-comparator-based method is more appropriate. However, a VBP framework that considers broad value elements of a drug, does not rely on comparators, and can be applied to the Japanese drug pricing system without limiting disease areas has not been proposed until now.

We developed the VBP framework “MARIE” (multiple criteria qualitative value-based pricing framework) as a methodology for calculating prices of new drugs applicable to the Japanese drug pricing system. This framework estimates new drug prices by qualitatively evaluating broader value elements without requiring comparative benchmarks [17]. In this method, twelve value elements are scored and a daily drug price is calculated based on these scores and the maximum number of patients using the drug. The reason for developing such a simplified method is that in Japan, drug pricing is listed within 60 to 90 days after new drug approval [18]. We believe that maintaining this speed contributes to sustainability of the universal health insurance system. Although the proposed pricing method was initially targeted with new drugs in mind, its design is highly versatile. It can be applied to drug prices for broad post-launch additions of new indications, including re-pricing for indicational change, re-pricing for change of dosage and administration, and re-pricing for market expansion. This makes it a potential solution for improving the problems associated with current re-pricing processes [19].

## 2. Materials and Methods

### 2.1. Overview of VBP Method

The MARIE method we developed is summarized as follows.

**Figure 1:**
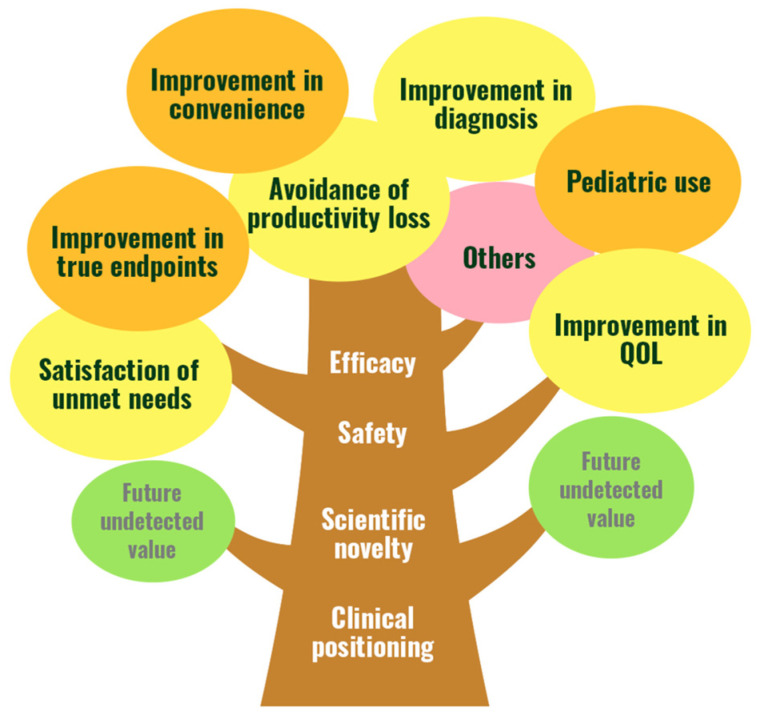
Value tree of the multiple criteria qualitative value-based pricing framework MARIE, MARIE, multiple criteria qualitative value-based pricing framework; QoL, quality of life. Adapted from Takami et al. [17].

The price is estimated as a daily drug price using points based on value elements and the maximum number of patients the drug will benefit. The value elements consist of base elements and additional elements. In our previous research, we considered a total of twelve elements: efficacy, safety, scientific novelty, and clinical positioning as base elements; and unmet needs, improvement in quality of life (QoL), improvement in true endpoints, avoidance of productivity loss, improvement in convenience, improvement in diagnosis, pediatric use, and others as additional elements (value tree, Figure 1). The detailed reasons for extracting these value elements are shown in our previous publication [17].

For base elements, efficacy was scored 20 points, while safety, scientific novelty, and clinical positioning were each scored 10 points, totaling 50 points (scoring criteria shown in Table 1). For additional elements, pediatric use and others were scored 10 points, while the others were scored 5 points. Points for each value element were assigned based on publicly available information, such as review reports and academic papers related to the target product.

Next, the daily drug price is calculated using a conversion table that base points and the maximum number of patients (Table 2, currency unit is JPY). The base drug prices in the conversion table were set in advance based on the daily drug prices listed in Japan over the past 10 years as drugs containing new active ingredients. To the base drug price obtained from base points and the maximum number of patients, an additional 2% premium is added for each additional point, yielding the final daily drug price. For example, if a drug earns 5 additional points, a 10% premium is applied to its base drug price. The maximum score for additional elements is set at 50 points, so the base price based on the basic elements is designed to be up to twice as much. Furthermore, this premium based on additional elements maintains continuity by following the approach of the current Japanese drug pricing system, such as the usefulness premium, marketability premium, and pediatric premium. The maximum score for additional elements is set at 50 points, so the base price based on the basic elements is designed to be up to twice as much. Furthermore, this premium based on additional elements maintains continuity by following the approach of the current Japanese drug pricing system, such as the usefulness premium, marketability premium, and pediatric premium.

### 2.2. Data Sources

The information sources used for the calculation were limited to publicly available information, as it was in our previous study. The data on drug prices at the time of listing and the maximum number of patients were extracted from the Central Social Insurance Medical Council materials [20].

### 2.3. Updated Conversion Table—Inclusion Criteria and Exclusion Criteria

Similar to our previous studies, in our analysis, we included drugs for which the daily drug price was explicitly stated in materials from the Central Social Insurance Medical Council, or drugs for which the daily drug price could be calculated based on their dosage and administration. We excluded the drugs that did not meet these criteria. Furthermore, drugs considered as a per-treatment drug price rather than a per-day drug price (Tremelimumab (Genetical Recombination), Ensitrelvir Fumaric Acid) as well as those drugs assessed as a per-season drug price (Nirsevimab (Genetical Recombination)) were rejected from inclusion. Excluded drugs in our previous study from FY 2012 to FY 2021 are 31 drugs (e.g., Lomitapide mesilate, Burosumab (genetic recombination)) as shown in Supplementary Table S1, so we extracted 364 drugs. Also, excluded drugs for the additional period from FY 2022 to FY 2024 in this analysis are 14 drugs as shown in Supplementary Table S2. As a result, we extracted 372 drugs. Since drugs with fewer patients tended to have higher daily drug prices, we summarized by descriptive statistics according to maximum patient category.

## 3. Results

For the 10-year period from FY2015 to FY 2024 (April 2015 to March 2025), we investigated and calculated the daily drug price at the time of listing for drugs newly added to the National Health Insurance Drug Price List as drugs that contained new active ingredients, and summarized the results using descriptive statistics. As in previous research, the conversion table created based on the median is shown in Table 3 (currency unit is JPY). The conversion table (Table 2) presented in our previous study covered the 10-year period from FY 2012 to FY 2021. Since new drug prices are listed annually in Japan, the table was updated with the latest information to ensure continuity with current drug prices. In this study as well, the median daily drug price demonstrated an increase as the maximum number of patients decreased.

## 4. Discussion

### 4.1. Significance of This Study

We updated the conversion table that monetizes base points calculated from value elements within the MARIE method, the framework we developed which qualitatively evaluates the various values of drugs. Since the number of drugs covered by the universal health insurance system increases annually, a system that reflects the latest prices benefits all stakeholders. Furthermore, as shown in our previous research, the true purpose of the MARIE method is to serve as a framework; a mechanism that can be updated to align with the healthcare environment and the times. In this study, we updated the conversion table that converts base points into monetary values based on the latest data.

### 4.2. Discussion of the Results

Analysis of drugs added in the National Health Insurance Drug Price List as containing new active ingredients during the ten-year period from FY 2015 to FY 2024 (April 2015 to March 2025) revealed the following trends compared to the data during the ten-year period from FY 2012 to FY 2021. First, in the category of more than 10,000 patients, the daily price remained largely unchanged. In the 3000 to 10,000 patient category, the daily drug price decreased by approximately 20%. Lastly, in the 1 to 10 patient category, the daily drug price increased by approximately 50%. While the trend of higher median daily drug prices with fewer maximum number of patients persists, drugs in the 1 to 10 patient category, which are ultra-orphan drugs, appear to have received higher drug price evaluations over the past three years.

## 5. Limitation

The conversion table for base points to monetary value (Table 3) was created using data from 372 drugs over the last ten years. While the trends did not differ significantly from those analyzed in our previous study, the values of the conversion table may vary depending on the reference period and the drugs included. The MARIE method primarily aims to propose a methodology for determining drug prices based on various values of the drug. Therefore, even if the values in the conversion table change, it is unlikely to significantly alter the methodological framework. Consequently, we consider the impact on the significance of our proposed method to be minor.

## 6. Conclusions

In the MARIE method, which qualitatively evaluates the various values of drugs we have developed, the conversion table that monetizes points calculated based on the elements of value plays a crucial role. Updating the conversion table based on the latest data maintains continuity with the current drug pricing system and is considered to contribute to the social implementation of the MARIE method. Furthermore, the true purpose of the MARIE method is to serve as a framework. By adapting the value elements and conversion tables to suit each country’s healthcare environment, it is hoped that the method will be implemented not only in developed countries but also in developing countries.

## Figures and Tables

**Table 1:** Scoring criteria for each value element.

Category	Value Elements	Points	Criteria
Base elements	Efficacy	20	Superiority over active comparator
		15	Non-inferiority over active comparator, superiority over placebo, and superiority as add-on therapy over standard of care alone
		10	Evaluated in a single-armed trial
	Safety	10	Acceptable, but with the description that information should be continuously gathered in post-marketing surveillance
		5	Acceptable, but with discussions of issues for warnings or contraindications in the review process for the approval
	Scientific novelty	10	Efficacy shown or expected to be shown + novel mechanism of action
		5	Efficacy shown or expected to be shown
	Clinical positioning	10	New or first treatment option
		5	One of several treatment options
Additional elements	Unmet needs	5	Treatment satisfaction is less than 50%, and drug contribution is less than 50%
		3	Treatment satisfaction is less than 50%, and drug contribution is more than 50% Treatment satisfaction is more than 50%, and drug contribution is less than 50%
		1	Treatment satisfaction is more than 50%, and drug contribution is more than 50%
	Improvement in QoL	5	Improvement from baseline or against placebo or an active comparator observed
		0	Improvement not observed or data not available
	Improvement in true endpoints	5	Improvement observed
		0	Improvement not observed or data not available
	Avoidance of productivity loss	5	Reports available on the productivity loss for the disease for which the drug was indicated
		0	No reports available on the productivity loss for the disease for which the drug was indicated
	Improvement in convenience	5	For example, self-administration possible, the administration interval extended, or the frequency of administration reduced
		0	None of above
	Improvement in diagnosis	5	Improvement observed
		0	Improvement not observed or data not available
	Pediatric use	10	Approved for use in children
		0	Not approved for use in children
	Others	10	If applicable
		0	If not applicable

Adapted from Takami et al. [17], QoL, quality of life.

**Table 2:** Conversion table of base drug prices according to base points and the maximum number of patients based on the data from FY2012~FY2021.

Base Points	Maximum Number of Patients														
	More Than 2,000,000	1,000,000 to 2,000,000	500,000 to 1,000,000	300,000 to 500,000	100,000 to 300,000	50,000 to 100,000	30,000 to 50,000	10,000 to 30,000	5000 to 10,000	3000 to 5000	1000 to 3000	500 to 1000	100 to 500	10 to 100	1 to 10
50	104	208	260	260	403	1209	1430	6500	13,130	14,040	27,560	30,030	56,680	66,560	120,250
45	96	192	240	240	372	1116	1320	6000	12,120	12,960	25,440	27,720	52,320	61,440	111,000
40	88	176	220	220	341	1023	1210	5500	11,110	11,880	23,320	25,410	47,960	56,320	101,750
35 *	80	160	200	200	310	930	1100	5000	10,100	10,800	21,200	23,100	43,600	51,200	92,500
30	72	144	180	180	279	837	990	4500	9090	9720	19,080	20,790	39,240	46,080	83,250
25	64	128	160	160	248	744	880	4000	8080	8640	16,960	18,480	34,880	40,960	74,000
20	56	112	140	140	217	651	770	3500	7070	7560	14,840	16,170	30,520	35,840	64,750
15	48	96	120	120	186	558	660	3000	6060	6480	12,720	13,860	26,160	30,720	55,500
10	40	80	100	100	155	465	550	2500	5050	5400	10,600	11,550	21,800	25,600	46,250
5	32	64	80	80	124	372	440	2000	4040	4320	8480	9240	17,440	20,480	37,000

Adapted from Takami et al. [17]. * To adjust the fractions of drug prices, the last digit was truncated when the median drug price at the time of launch corresponding to 35 points had a total of 3 digits, and the last 2 digits were truncated when the median drug price at the time of launch corresponding to 35 points had a total of 4 digits or more. Based on these values, the table was created by extrapolation according to the following criteria: when the score increased by 5 points, the drug price was increased by 10%, and when the score decreased by 5 points, the drug price was decreased by 10%.

**Table 3:** Conversion table of base drug prices according to base points and the maximum number of patients based on the data from FY2015~FY2024.

Base Points	Maximum Number of Patients														
	More Than 2,000,000	1,000,000 to 2,000,000	500,000 to 1,000,000	300,000 to 500,000	100,000 to 300,000	50,000 to 100,000	30,000 to 50,000	10,000 to 30,000	5000 to 10,000	3000 to 5000	1000 to 3000	500 to 1000	100 to 500	10 to 100	1 to 10
50	104	260	260	273	585	1287	1560	6500	10,920	11,050	31,070	37,310	70,070	66,560	183,430
45	96	240	240	252	540	1188	1440	6000	10,080	10,200	28,680	34,440	64,680	61,440	169,320
40	88	220	220	231	495	1089	1320	5500	9240	9350	26,290	31,570	59,290	56,320	155,210
35 *	80	200	200	210	450	990	1200	5000	8400	8500	23,900	28,700	53,900	51,200	141,100
30	72	180	180	189	405	891	1080	4500	7560	7650	21,510	25,830	48,510	46,080	126,990
25	64	160	160	168	360	792	960	4000	6720	6800	19,120	22,960	43,120	40,960	112,880
20	56	140	140	147	315	693	840	3500	5880	5950	16,730	20,090	37,730	35,840	98,770
15	48	120	120	126	270	594	720	3000	5040	5100	14,340	17,220	32,340	30,720	84,660
10	40	100	100	105	225	495	600	2500	4200	4250	11,950	14,350	26,950	25,600	70,550
5	32	80	80	84	180	396	480	2000	3360	3400	9560	11,480	21,560	20,480	56,440

* To adjust the fractions of drug prices, the last digit was truncated when the median drug price at the time of launch corresponding to 35 points had a total of 3 digits, and the last 2 digits were truncated when the median drug price at the time of launch corresponding to 35 points had a total of 4 digits or more. Based on these values, the table was created by extrapolation according to the following criteria: when the score increased by 5 points, the drug price was increased by 10%, and when the score decreased by 5 points, the drug price was decreased by 10%.

## Data Availability

The original contributions presented in this study are included in the article/Supplementary Materials. Further inquiries can be directed to the corresponding author.

## Supplementary Materials

The following supporting information can be downloaded at: https://www.mdpi.com/article/10.3390/jmahp14010012/s1, Table S1: Active product ingredients excluded in summarizing initial daily drug prices at the time of launch from FY2012 to FY2021; Table S2: Active product ingredients excluded in summarizing initial daily drug prices at the time of launch from FY2022 to FY2024.
